# Evaluation of *Funneliformis mosseae* inoculation effects on growth, nutrient uptake, and essential oil content in Turkish oregano under drought stress

**DOI:** 10.7717/peerj.19499

**Published:** 2025-06-19

**Authors:** Mina Najafi, Burçin Çokuysal, Younes Rezaee Danesh, Beatrice Farda, Amedeo Mignini, Marika Pellegrini

**Affiliations:** 1 Department of Soil Science and Plant Nutrition, Faculty of Agriculture, Ege University, Izmir, Turkey; 2 Department of Plant Protection, Faculty of Agriculture, Van Yuzuncu Yil University, Van, Turkey; 3 Department of Life, Health and Environmental Sciences, University of L’Aquila, L’Aquila, Italy

**Keywords:** *Origanum onites* L., Arbuscular Mycorrhizal Fungi, Abiotic stress tolerance, Sustainable agriculture, Plant biostimulants

## Abstract

**Background:**

Turkish oregano (*Origanum onites* L.) is a perennial herb widely recognized for its medicinal, cosmetic, and culinary uses due to its antioxidant and antimicrobial properties. Drought is a significant stressor for crops, particularly affecting *O. onites* quality and yield. Arbuscular mycorrhizal fungi (AMF) establish symbiotic relationships with plant roots, enhance plant growth, and improve tolerance to abiotic stresses such as drought.

**Methods:**

This study investigates the effects of *Funneliformis mosseae* inoculation on *O. onites* growth, nutrient content, and essential oil yield under varying drought conditions. A factorial experiment was conducted with eight treatments, consisting of two factors: irrigation levels (100%, 75%, 50%, and 25%) and AMF inoculation (with and without). The experimental design was completely randomized with three replicates.

**Results:**

Results demonstrated that AMF inoculation significantly improved the fresh and dry weight of *O. onites* compared to non-inoculated controls (+11% and +16%, respectively). Moreover, AMF-inoculated plants showed notable increases in potassium (+7%) and nitrogen (+12%) contents. The essential oil yield was also significantly higher in AMF-inoculated plants (+3%). Increasing water stress levels significantly decreased the number of AMF spores (−47%) and the percentage of fungal colonization (−57%). Nevertheless, under drought stress mycorrhizal inoculation significantly maintained plant biomass and nutrient uptake comparable to full irrigation. The AMF drought tolerance effects were confirmed at 75%, 50%, and 25% irrigation rates.

## Introduction

The Mediterranean area displays unique biological and ecological diversity, higher than in other countries in the temperate climate zone. Taxa belonging to the Lamiaceae are widely distributed in this area, thriving in various habitats and altitudes (Xu & Chang, 2017). Approximately 250 genera and over 7,000 species are included in this family. Among genera, *Salvia*, *Scutellaria*, *Stachys*, *Plectranthus*, *Hyptis*, *Teucrium*, *Origanum, Thymus*, *Vitex*, and *Nepeta* are necessary for their medicinal and food uses (Bekut et al., 2018) and belong to the medicinal aromatic plants (MAPs) group (Ramos da Silva et al., 2021).

Species belonging to MAPs are highly valued for their various plant parts (mainly leaves and flowers), which contain a wide range of secondary metabolites (Rasool Hassan, 2012; Chouhan, Sharma & Guleria, 2017; Raveau, Fontaine & Sahraoui, 2020). MAPs are also widely used as spices, flavoring agents, and in the cosmetic and pharmaceutical industries (Sofowora, Ogunbodede & Onayade, 2013; Samarth, Samarth & Matsumoto, 2017). Their essential oils (EOs), known for their antimicrobial, antioxidant, and antibacterial effects, have garnered increasing attention for their potential health benefits (Dhifi et al., 2016; Li et al., 2020; Mugao et al., 2020). Therefore, the cultivation of MAPs is an essential source of effective healthcare products, supporting the health and wellness of 80% of the global population (Karunamoorthi et al., 2013; Ekor, 2014).

For these reasons, the demand for MAPs has grown considerably over the past three decades (Hamilton, 2004). However, global warming and associated climatic changes such as droughts, heat, salinity, and flooding are posing significant challenges to the growth, development, and productivity of MAPs (Crockett & Westerling, 2018; Zandalinas et al., 2018). MAPs are particularly susceptible to these adverse environmental conditions, which can severely impair their productivity (Rahman, Iqbal & Husen, 2023).

Among MAPs threatened species, Turkish oregano (*Origanum onites* L.) has a relevant ecological role. This species is primarily found in Sicily, Greece, and Türkiye, and is cultivated in regions such as Balıkesir, Manisa, Uşak, İzmir, Muğla, Aydın, Denizli, Antalya, and Isparta (http://www.worldfloraonline.org/taxon/wfo-0000260691, accessed on 29 October 2024). This species is flagged as endangered, and it is listed in the Red List of Flora of Italy Portal—Resources from Acta Plantarum (https://dryades.units.it/floritaly/index.php?procedure=taxon_page&tipo=all&id=4615, accessed on 15 November 2024). Drought is the primary stressor that negatively impacts *O. onites* distribution, quality, and yield of shrubs and their EOs contents (Stefanakis et al., 2024).

Plant drought stress tolerance can be increased by arbuscular mycorrhizal fungi (AMF) (Arpanahi et al., 2020; Eshaghi Gorgi et al., 2022), which establish symbiotic relationships with plant roots (Zou, Wu & Kuča, 2021). The application of AMF contributes to this resilience through various mechanisms, including direct water uptake, enhancement of antioxidative defenses, modulation of hormonal balance, induction of morphological changes, transcriptomic regulation, and improvement of soil physical and chemical properties (Boorboori & Lackóová, 2025). Although prolonged and severe drought stress negatively affects both symbiotic partners, the overall benefits of this association greatly surpass the associated costs (Das & Sarkar, 2024). Therefore, understanding the dynamics and mechanisms of the plant-AMF interaction under drought stress is essential for developing sustainable agricultural practices and drought mitigation strategies.

No previous studies investigated the AMF contribution to *O. onites* drought stress tolerance. Conversely, numerous studies have demonstrated that AMF inoculation increases MAPs tolerance to abiotic stresses, such as drought, salinity, and pollution, through various morphological, physiological, and molecular mechanisms (Abd_Allah et al., 2015; Hazzoumi et al., 2015; Pedranzani et al., 2016; Xie et al., 2018; Bitterlich et al., 2018; Amanifar & Toghranegar, 2020; Ebrahimi et al., 2021; Cheng et al., 2021). Therefore, we hypothesized that AMF application on *O. onites* would effectively improve water stress resistance. This study tested the validity of our hypothesis by examining the effects of *Funneliformis mosseae* AMF inoculation on plant growth, mineral element content, and EOs yield of *O. onites* under drought stress.

## Materials & Methods

### Materials

Seeds of *Origanum onites* L. were sourced from the Aegean Agricultural Research Institute, Türkiye. The arbuscular mycorrhizal fungus, *F. mosseae*, was selected for its broad adaptability and efficiency in plant symbiosis. *F. mosseae* was obtained from the Department of Plant Protection, Faculty of Agriculture, Van Yüzüncü Yıl University, Türkiye. The AMF inoculum had a concentration of 168 spores per gram of soil.

### Greenhouse experimental setup

**Figure 1:**
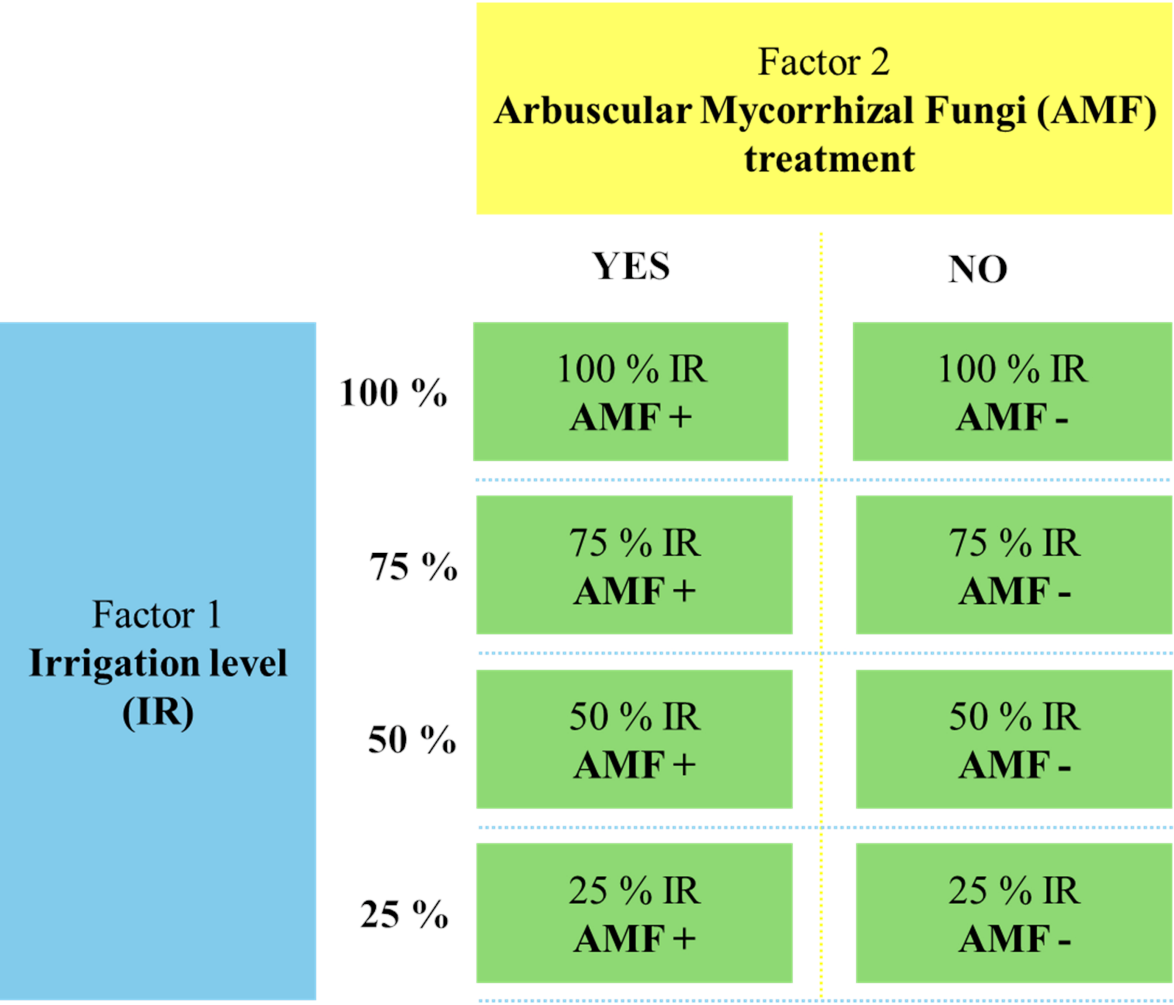
The flowchart of the experimental setup.

The greenhouse experiment was conducted from 2020 to 2022 at the Department of Soil Science and Plant Nutrition, Faculty of Agriculture, Ege University, Türkiye. The study followed a factorial design, incorporating two main factors: drought stress (four irrigation levels: 25%, 50%, 75%, 100%) and AMF treatment (AMF+ with mycorrhiza, AMF- without mycorrhiza). A completely randomized design was employed with three replicates, resulting in 24 pots. The experimental treatments are displayed in Fig. 1. Each pot (400 g capacity) was filled with 320 g of sterilized sandy soil (texture: 65.5% sand, 31.0% silt, and 3.5% clay, pH: 8.5, organic matter content: 1.5%) and 80 g of AMF inoculum for the AMF+ treatments. In AMF-treatments, pots were filled with 400 g of sterilized soil without AMF inoculum. Five seeds of *O. onites* were sown in each pot, and the plants were grown under controlled greenhouse conditions for 90 days (24 ± 1 °C, 60% relative humidity, 16 h light/8 h dark cycle). Drought stress was imposed from the time of planting onwards, according to the designated irrigation levels, and plants were fertilized weekly using a Hoagland solution (without phosphorus). After 90 days, plants were harvested for the analyses detailed below. Soil and root samples were stored at 4 °C and −20 °C for subsequent AMF spore counts and root colonization analysis.

### Plant growth parameters

Plant fresh weights (PFW) were recorded after the plants were washed. Plant dry weights (PDW) were recorded after drying at 65 °C until constant weight (after approximately 96 h). The dried matrix was ground into powder to be analyzed for nutrient content.

### Plant EOs yield

The EO content of the dried plant samples was measured volumetrically using a Clevenger apparatus. Thirty grams of dried plant material were placed in a 1,000 mL glass flask containing 300 mL of distilled water. The mixture was distilled for 4 h. The plant EOs yields have been calculated (EOs % = g EOs/g dried matrix x 100) and were expressed as a percentage of the sample’s dry weight (Palmieri et al., 2021).

### Nutrient contents

Total nitrogen (N) content was determined using the Kjeldahl method (Kirk, 1950). For the analysis of phosphorus (P), potassium (K), calcium (Ca), sodium (Na), magnesium (Mg), and iron (Fe) contents, plant samples were digested with a 1:4 mixture of hydrochloric acid and perchloric acid. Phosphorus was quantified using the vanadomolybdate yellow colorimetric method, potassium by flame photometry, and magnesium and iron by atomic absorption spectrophotometry (Kacar & Inal, 1972; Kacar & Inal, 2008; Kacar & Kovancı, 1982).

### AMF spore numbers and root colonization

The impact of drought stress on AMF spore numbers and root colonization was evaluated. AMF spores were extracted from 10 g soil samples using the ultrasound centrifuge technique (Boyno et al., 2023) and microscope observation for spore counting. Root samples (∼0.5 g) were washed with distilled water, stained with 0.05% trypan blue, and de-stained with lactic acid. The roots were cut into 1–2 cm segments. The mycorrhizal density (M%) and frequency (F%) were calculated as previously described (Demir et al., 2024).

### Statistical analysis

For each parameter, the data from all five plants within each block was collected and used to calculate the block average value used in the statistical analyzes. All data are expressed as the average value across all blocks. Data were analyzed using Python (version 3.11). The normality of the data was assessed using the Shapiro–Wilk test (SciPy library, version 1.10.1). A two-way ANOVA was conducted using the Statsmodels library (version 0.13.5) for variables determined to be normally distributed. Non-normally distributed variables were analyzed using the Scheirer–Ray–Hare test. *Post hoc* analyses were applied where appropriate, including Fisher’s Least Significant Difference (LSD) test for ANOVA results (*via* Statsmodels) and the Conover-Iman test for Kruskal–Wallis results (SciPy). Correlation and principal component analyses were performed to investigate variable correlations. Visualizations were performed using Matplotlib (version 3.7.1), while data were managed using Pandas (version 1.5.3).

## Results

Descriptive statistics of the dataset are presented in Table 1. Plants displayed a mean PFW of 19.1 g and a PDW of 4.7 g, with moderate variability in PFW and lower variability in PDW. A low to moderate variability was observed for EOs contents. Plant nutrient analysis showed moderate variability in nitrogen (N) and potassium (K); phosphorus (P) and calcium (Ca) displayed low variability. Magnesium (Mg) was consistent across samples, while iron (Fe) and sodium (Na) contents varied substantially. Fungal colonization (F) and spore numbers (SN) showed significant variability. Based on the Shapiro–Wilks normality test, variables displayed a Normal distribution except for EOs and iron contents, fungal colonization, and spore numbers.

**Table 1:** Descriptive statistics and Shapiro Wilks results.

	Plant FW (g)	Plant DW (g)	EOs (%)	Fe (mg/kg)	K (mg/kg)	Ca (mg/kg)	P (mg/kg)	N (mg/kg)	Mg (mg/kg)	Na (mg/kg)	F (%)	Spore no. (no spore/ 10 g soil)
mean	19.12	4.69	2.65	626.67	3.45	1.10	0.37	1.88	0.36	2,775.00	28.18	213.7
std	3.44	0.85	0.41	168.63	0.49	0.17	0.04	0.39	0.04	273.86	14.37	85.5
min	12.61	3.12	2.12	353.00	2.45	0.80	0.29	1.30	0.26	2,300.00	13.30	100.0
max	24.66	6.98	3.44	854.00	4.30	1.40	0.45	2.57	0.44	3,200.00	52.30	335.0
W-value	0.96	0.96	0.90	0.90	0.96	0.96	0.97	0.93	0.97	0.94	0.79	0.8
*p*-value	0.35	0.43	0.02	0.02	0.48	0.46	0.78	0.11	0.62	0.21	<0.01	<0.01

**Notes.**

FW Fresh weight DW Dry weight Eos Essential oils K Potassium Ca Calcium P Phosphorous N Nitrogen Mg Magnesium Na Sodium Fe Iron F Fungal colonization

### Plant growth parameters

**Figure 2:**
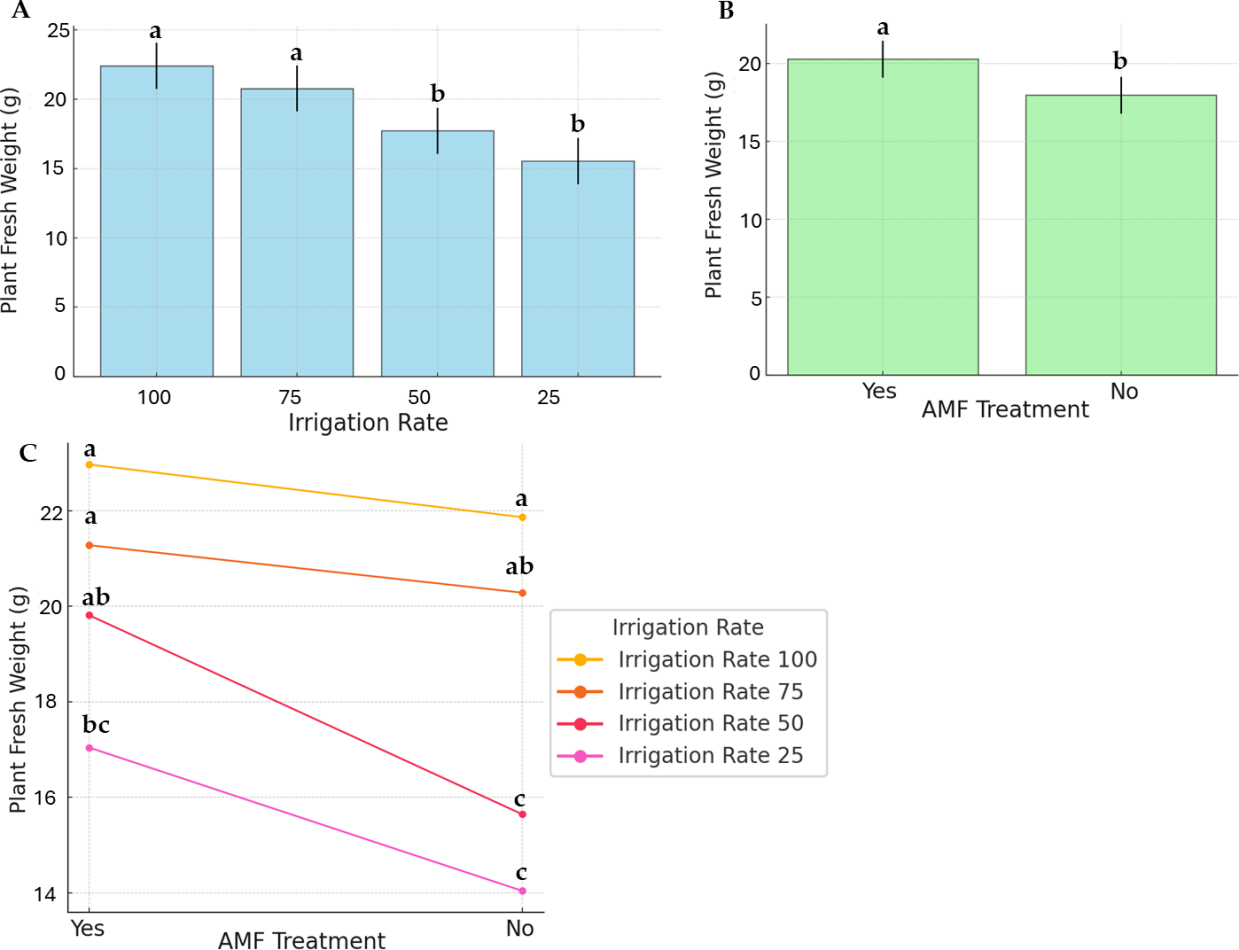
Effect of irrigation rate (A), AMF treatment (B), and their interaction (C) on plant fresh weight.

Figure 2 displays PFW and the effects of irrigation rate, AMF treatment, and their interaction. The highest and lowest average PFW (22.97 g and 14.04 g, respectively) were recorded at 100% irrigation with AMF inoculation and 25% irrigation without AMF inoculation. For plants without AMF inoculation, irrigation levels significantly impacted fresh weight (Fig. 2B). Specifically, the highest fresh weights were achieved at 100% and 75% irrigation, whereas the lowest were observed at 25% and 50% irrigation. In contrast, AMF-inoculated plants exhibited no significant differences in fresh weight across irrigation levels. Comparisons of treatments revealed that AMF-inoculated plants had higher fresh weights than non-inoculated ones.

**Figure 3:**
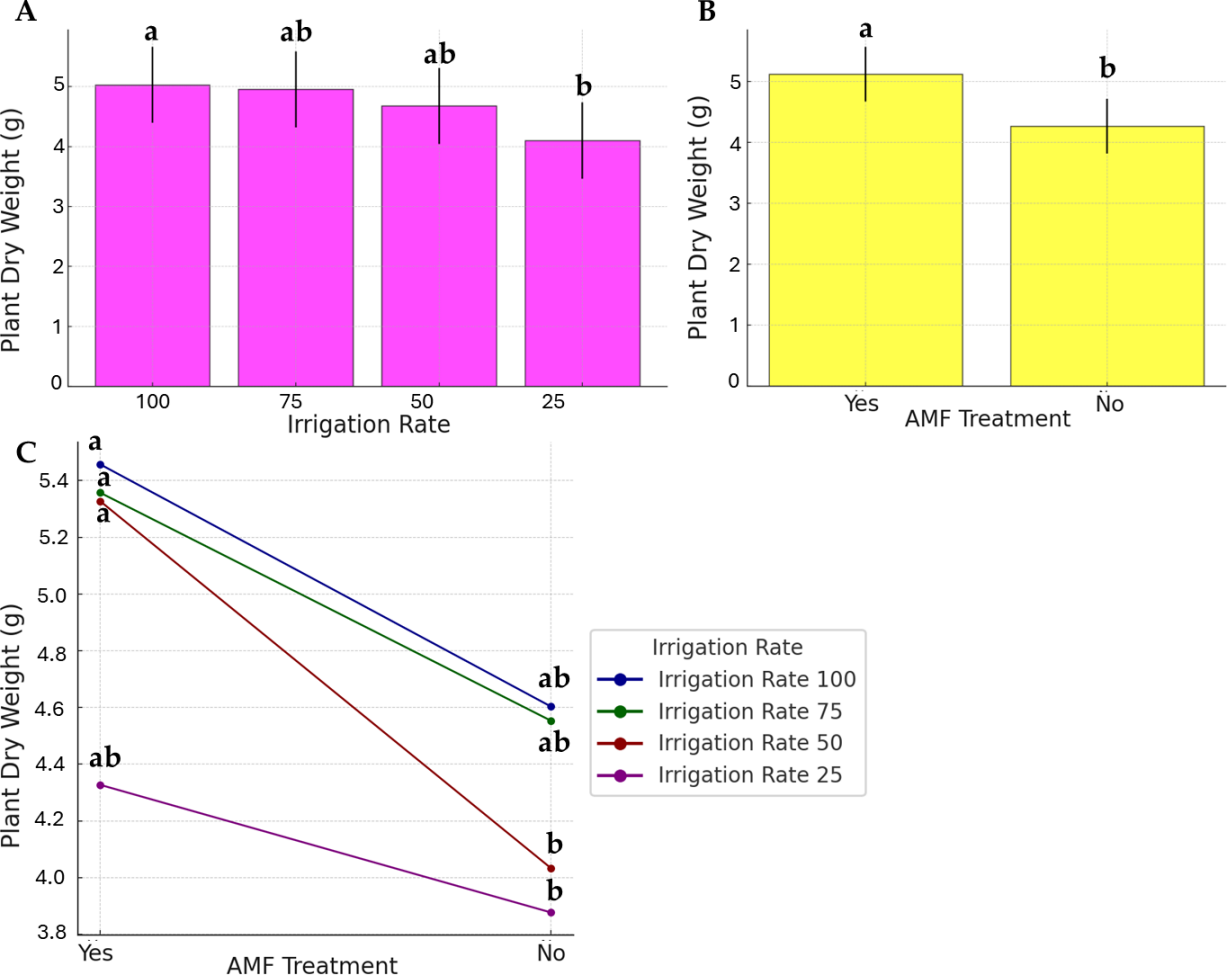
Effect of irrigation rate (A), AMF treatment (B), and their interaction (C) on plant dry weight.

As presented in Fig. 3, the water stress, AMF application, and their interaction effects were evident in plant dry weight, even if to a lesser extent. Data on the irrigation rate were separated into two distinct groups; the highest dry weight was observed at 100% irrigation rate (group a), while the lowest was at 25% (group b). No significant differences were observed at 50% and 75%, and they were found similar to the a and b statistical groups (group ab). AMF treatment was found to be significant. However, its contribution to the factor’s interaction was low (Fig. 3C).

### Plant EOs yield

**Figure 4:**
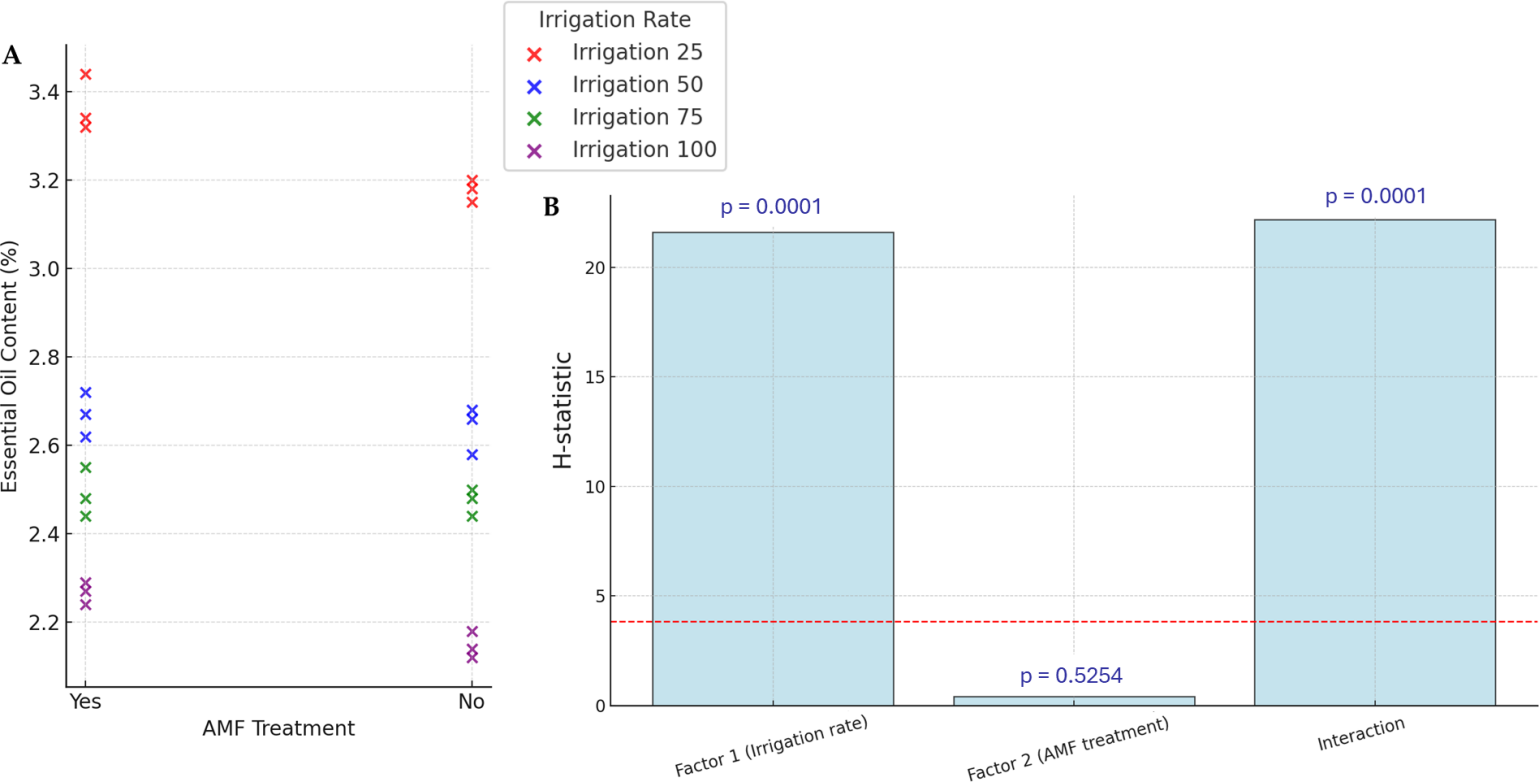
Effect of irrigation rate, AMF treatment and their interaction on EOs content. (A) Scatter plot of EOs contents in the different experimental conditions. (B) Bar plots summarizing the Scheirer-Ray-Hare test results for EOs content. Bars represent H-statistic values for Factor 1 (Irrigation rate), Factor 2 (AMF treatment), and their interaction. The dashed red line indicates the significance threshold (*p* = 0.05), *p*-values are annotated above each bar.

The yields of EOs are presented in Fig. 4. Accumulation of EOs in plants increased significantly with lower irrigation rates (*p* < 0.05), with the highest values (34.4%) observed in the lower irrigation rate with AMF treatment. However, the AMF treatment did not significantly affect the content of EOs (*p* > 0.05). The lowest value in EOs content is observed in the higher irrigation rate with no AMF treatment (21.2%). The interaction Irrigation rate × AMF treatment was found to be significant for this parameter.

### Nutrient contents

**Figure 5:**
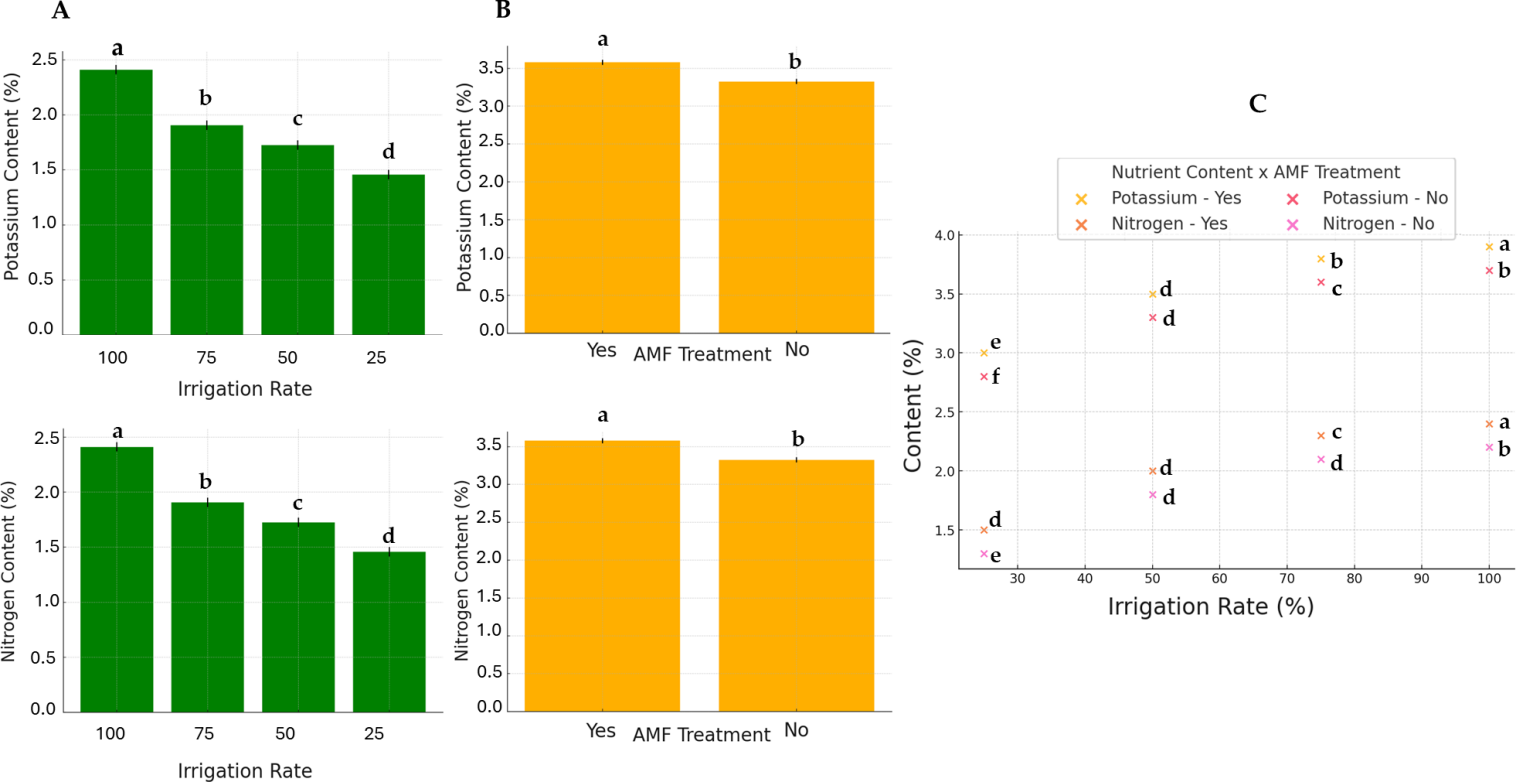
Effect of irrigation rate (A), AMF tretment (B) and their interaction (C) on plant potassium and nitrogen contents.

**Figure 6:**
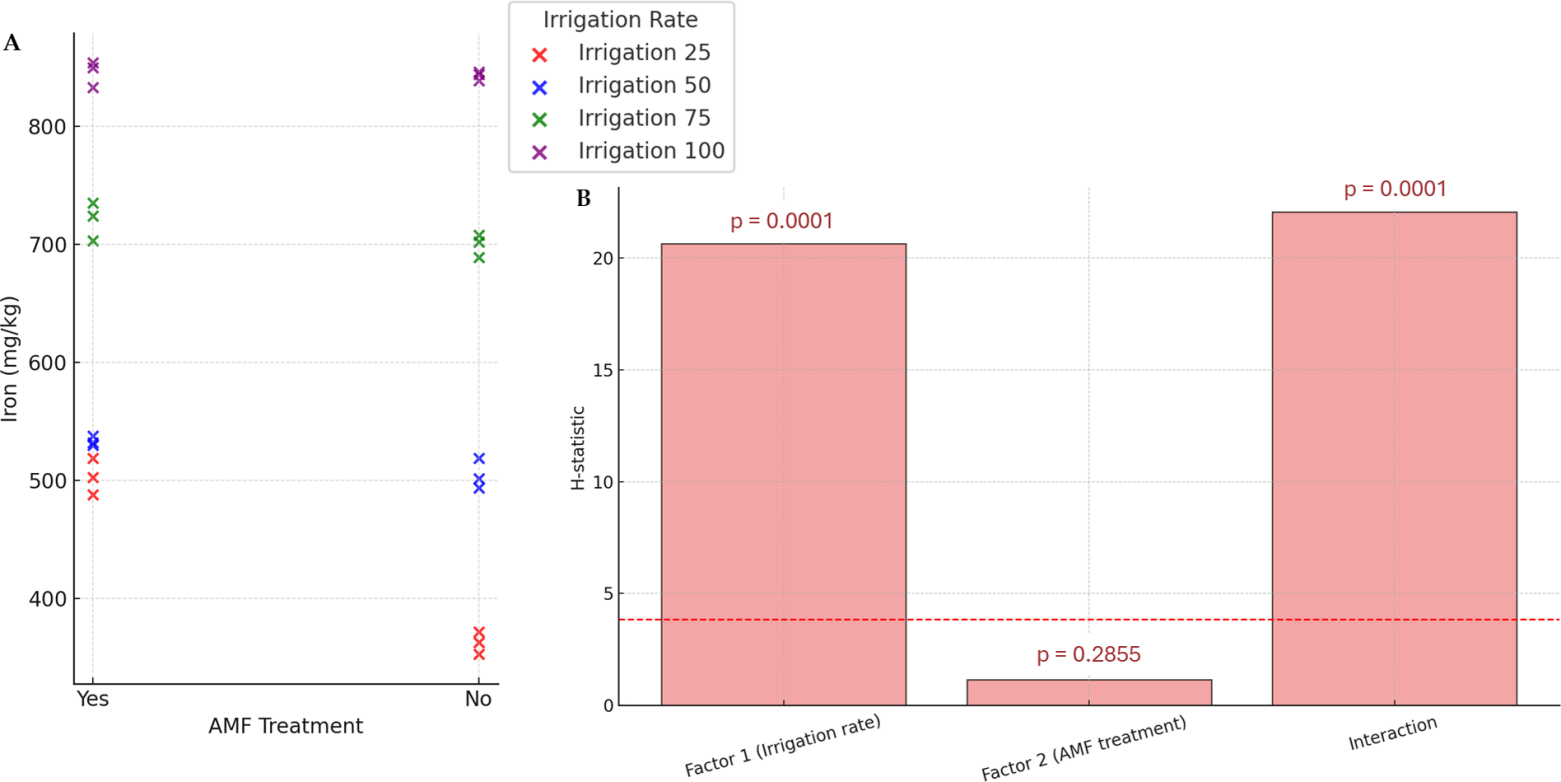
Effect of irrigation rate, AMF treatment and their interaction on iron content. (A) Scatter plot of iron contents in the different experimental conditions. (B) Bar plots summarizing the Scheirer-Ray-Hare test results for iron content. Bars represent H-statistic values for Factor 1 (Irrigation rate), Factor 2 (AMF treatment), and their interaction. The dashed red line indicates the significance threshold (*p* = 0.05), *p*-values are annotated above each bar.

Generally, except for K and N, the AMF treatment did not significantly affect nutrients. Conversely, the irrigation rate effect was found to be significant on all parameters. The interaction between these two factors also significantly influenced nutrient contents. The results of N and K contents are presented in Fig. 5. The highest K and N contents were found at a 100% irrigation rate in the presence of AMF inoculation (4.13% and 2.55%, respectively). The lowest contents were observed at 25% irrigation without AMF inoculation (2.55% and 1.31%, respectively). The results of Fe contents (not normally distributed) are presented in Fig. 6. According to Scheirer-Ray-Hare test results, irrigation impacted Fe concentration significantly, but not by the AMF treatment (*p* > 0.05). However, the interaction between irrigation rate and AMF treatment was shown to be significant. At lower irrigation rates, plants’ Fe accumulation decreased significantly (*p* < 0.05). The highest Fe content was observed at 100% irrigation, while the lowest was at 25%. The effects on the other nutrients’ contents are summarized in Table 2. The highest irrigation rate for Ca, K, and Mg promoted the best nutrient accumulation in plants, both in the presence and absence of AMF. The lowest contents were observed at 25% of irrigation. The significant effect of AMF treatment was only found for magnesium (Mg) (*p* < 0.05). Water stress led to an increase in Na accumulation. Due to the accumulation of salts during water stress, this opposite trend allowed us to separate irrigation levels into two statistical groups, *i.e.,* 25–50% and 75–100% groups. At the same time, AMF treatment had no significant effects. Also, the interaction irrigation rate x AMF was significant in this case.

**Table 2:** Effects of irrigation levels, AMF treatment and their interaction on nutritient contents.

IR	AMF	Ca (%)		P (%)		Mg (mg/Kg)		Na (mg/Kg)	
25	+	0.88	d	0.347	cd	0.347	c	3,066.67	a
	−	0.90	cd	0.310	d	0.290	d	3,100.00	ab
mean		0.89	C	0.328	C	0.318	C	3,083.33	A
50	+	1.03	bcd	0.373	bc	0.357	abc	3,000.00	ab
	−	1.10	abc	0.370	bc	0.353	bc	2,833.33	bc
mean		1.07	B	0.372	B	0.355	B	2,916.67	A
75	+	1.20	ab	0.393	ab	0.370	abc	2,533.33	cd
	−	1.22	ab	0.383	abc	0.373	abc	2,633.33	d
mean		1.21	AB	0.388	AB	0.372	AB	2,583.33	B
100	+	1.23	ab	0.420	a	0.400	a	2,500.00	d
	−	1.27	a	0.390	ab	0.397	ab	2,533.33	d
mean		1.25	A	0.405	A	0.398	A	2,516.67	B
LSD-value	IR	0.14^*^		0.03^*^		0.03^*^		181.88^*^	
	AMF	0.10^ns^		0.02^ns^		0.02^ns^		128.61^ns^	
	IR X AMF	0.20^*^		0.04^*^		0.05^*^		257.22^*^	

**Notes.**

IR Irrigation rate AMF AMF treatment

* Significant at *p* < 0.05.

ns Not significant.

Different letter casing indicates significantly different results (Fisher LSD *post hoc* test, *p* < 0.05). Lowercase letters, IR × AMF interaction; uppercase letter, IR factor.

### AMF spore numbers and root colonization

**Figure 7:**
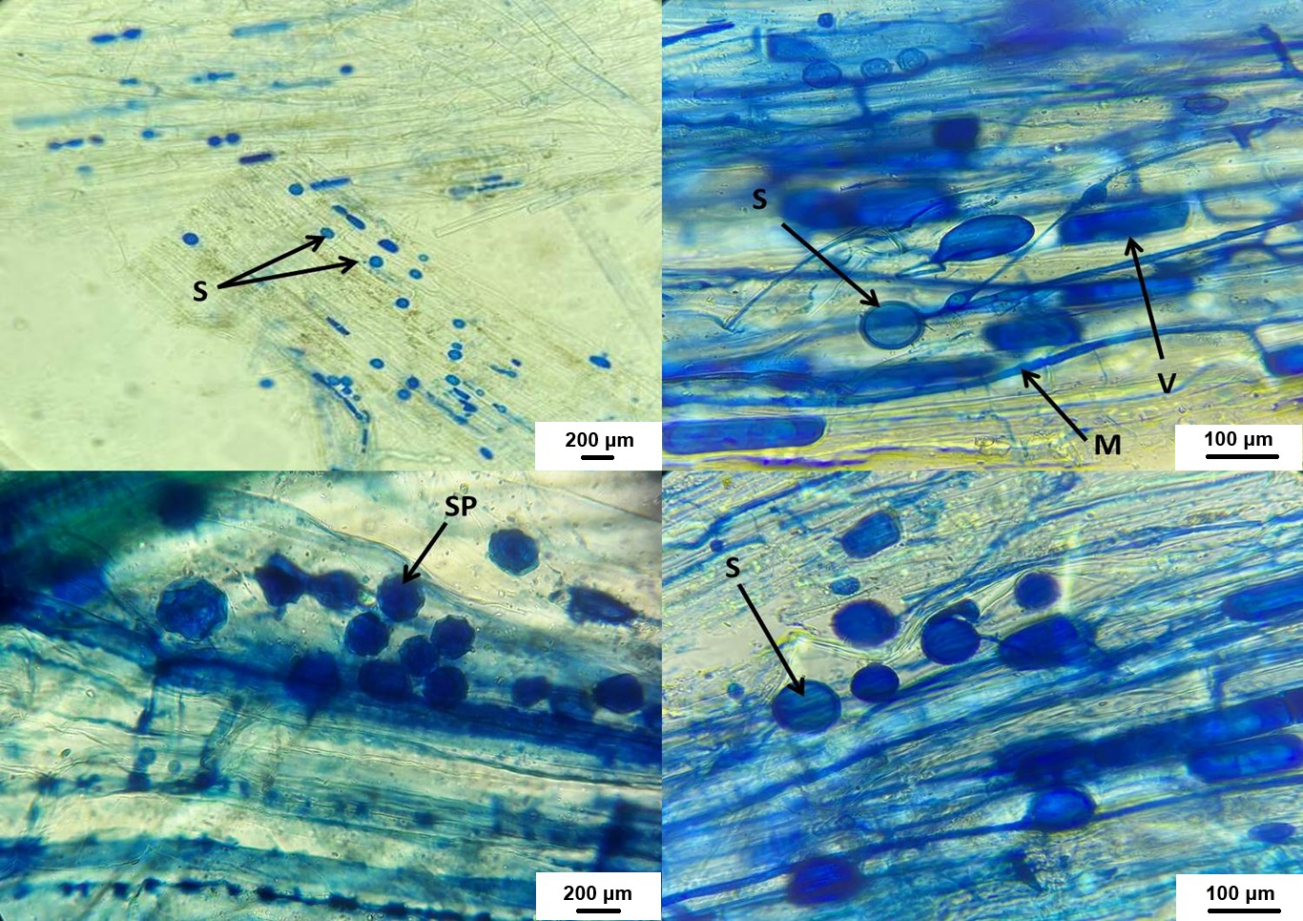
AMF structures in host plant roots. V, vesicles; M, fungus mycelium, S, spores; SP, sporocarps.

**Figure 8:**
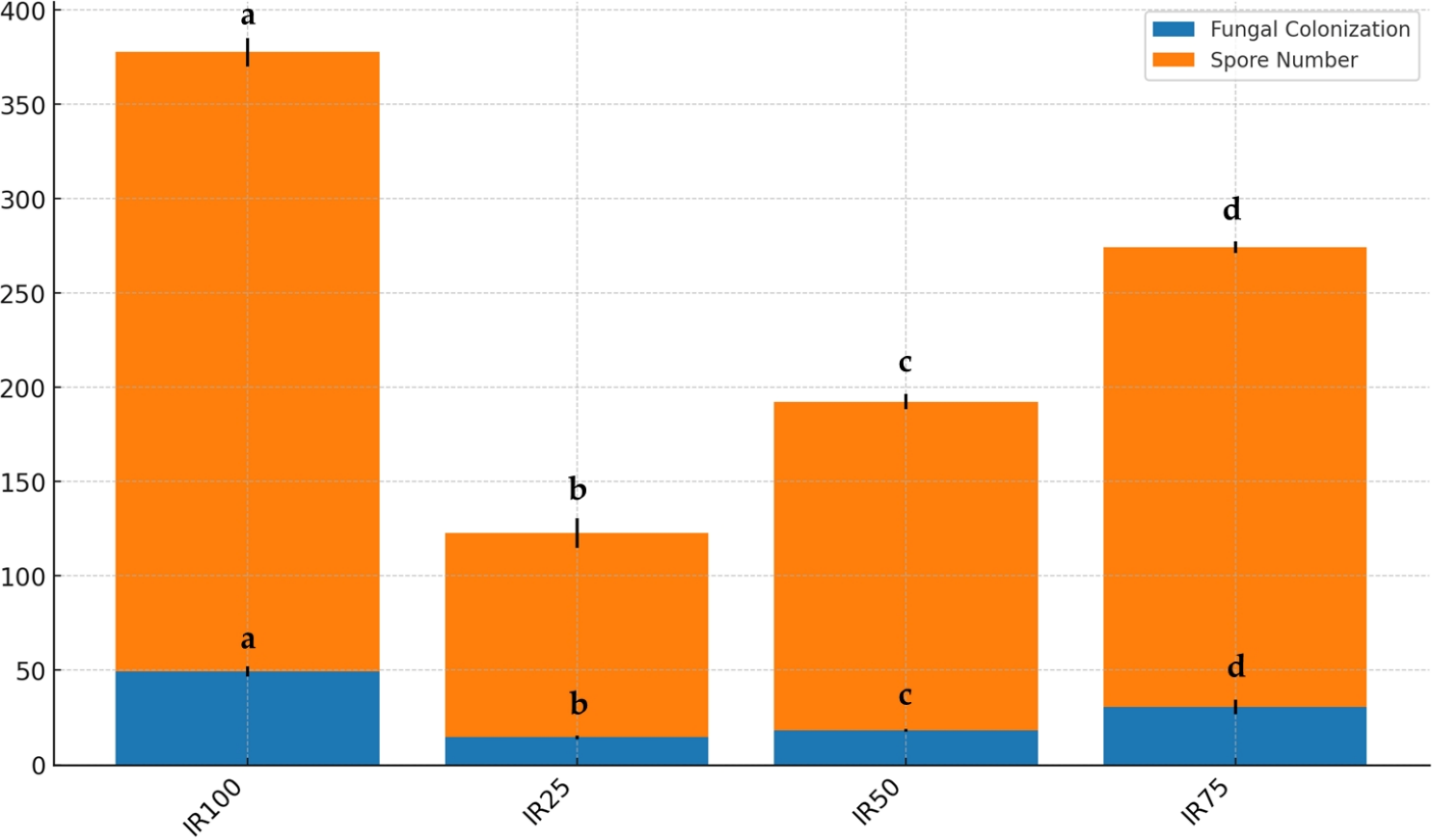
Stacked bar plot of fungal colonization (%) and spore number at different irrigation rates (IR25, IR50, IR75, IR100). The blue bars represent the mean fungal colonization, while the orange bars represent the mean spore number. Error bars indicate the standard deviation for each variable. Results followed by different case letters are significantly different according to Kruskal–Wallis followed by Conover-Iman *post hoc* test (*p* < 0.05).

As shown in Fig. 7, AMF structures were identified in all root segments, confirming colonization in all host plants. Various AMF structures, including extra- and intra-radical mycelia, vesicles, spores, and sporocarps, were observed, with their density varying among samples. F and SN data are displayed in Fig. 8. Water stress significantly reduced AMF spore numbers and colonization rates (*p* < 0.05). The highest spore numbers (328.33 spores per 10 g of soil) and the highest colonization index (49.33%) were recorded at 100% irrigation. The lowest values (108.33 spores per 10 g of soil and 14.50%, respectively) were found at 25% irrigation (*p* < 0.05).

### Correlations and principal components analysis

**Figure 9:**
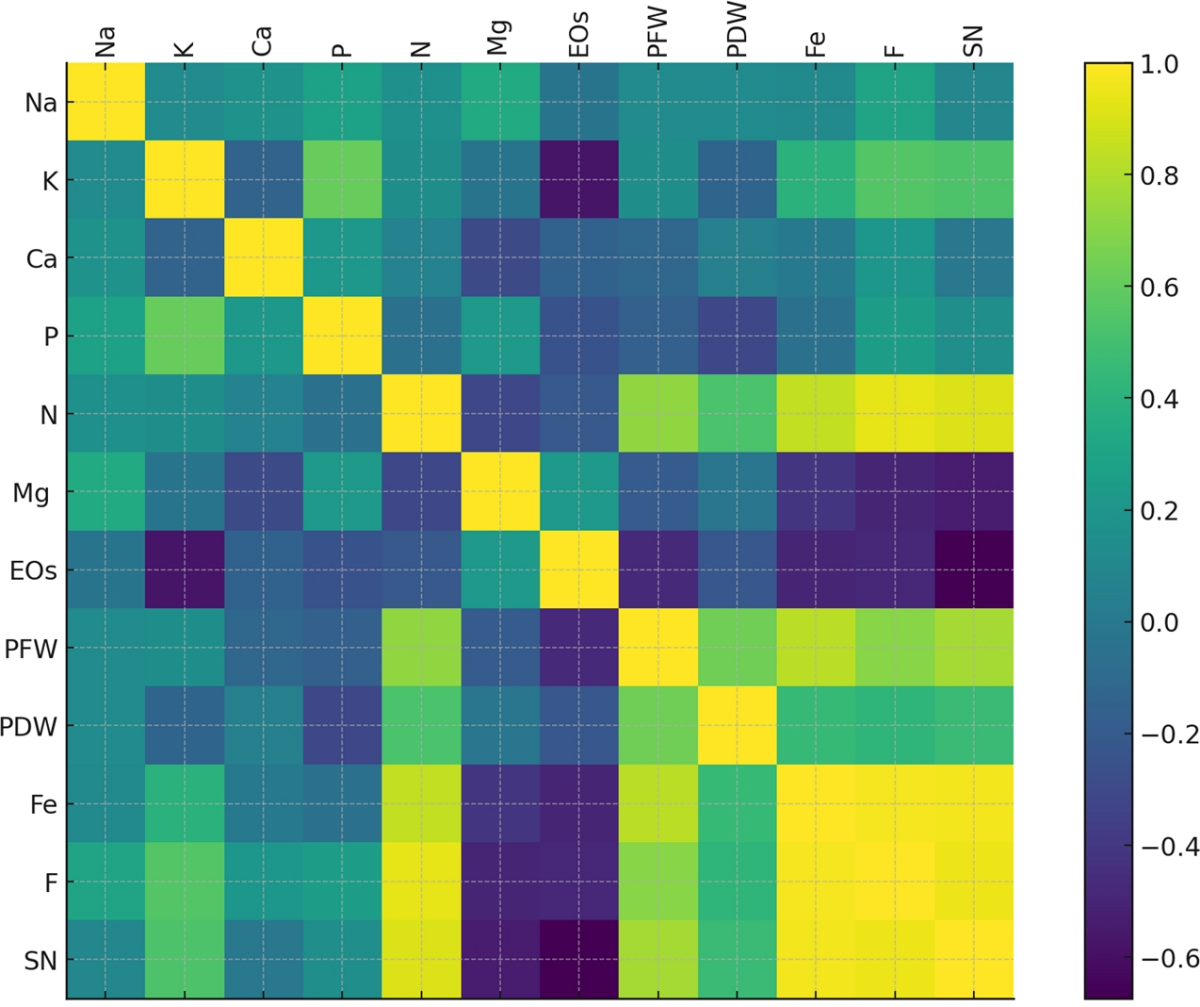
Heatmap of the correlation matrix displaying relationships between plant growth parameters, nutrient contents, fungal colonization (F), and spore number (SN). The color gradient from blue to green to yellow represents the strength and direction of correlations, with darker shades indicating stronger relationships.

To highlight the intricate relationships between plant traits, nutrient dynamics, and treatments, a correlation analysis was carried out. Figure 9 depicts the heatmap obtained. PFW and PDW (highly positively correlated to each other) correlated positively with macronutrients such as N, P, and K. Ca and Mg showed moderate correlations. At the same time, Fe appears linked to AMF treatment (F and SN), directly supporting plant growth (PFW and N). EOs, in contrast, are inversely correlated with biomass and nutrient uptake. Similarly, fungal colonization and spore number exhibited positive correlations with nutrient absorption variables like N, P, and K.

**Figure 10:**
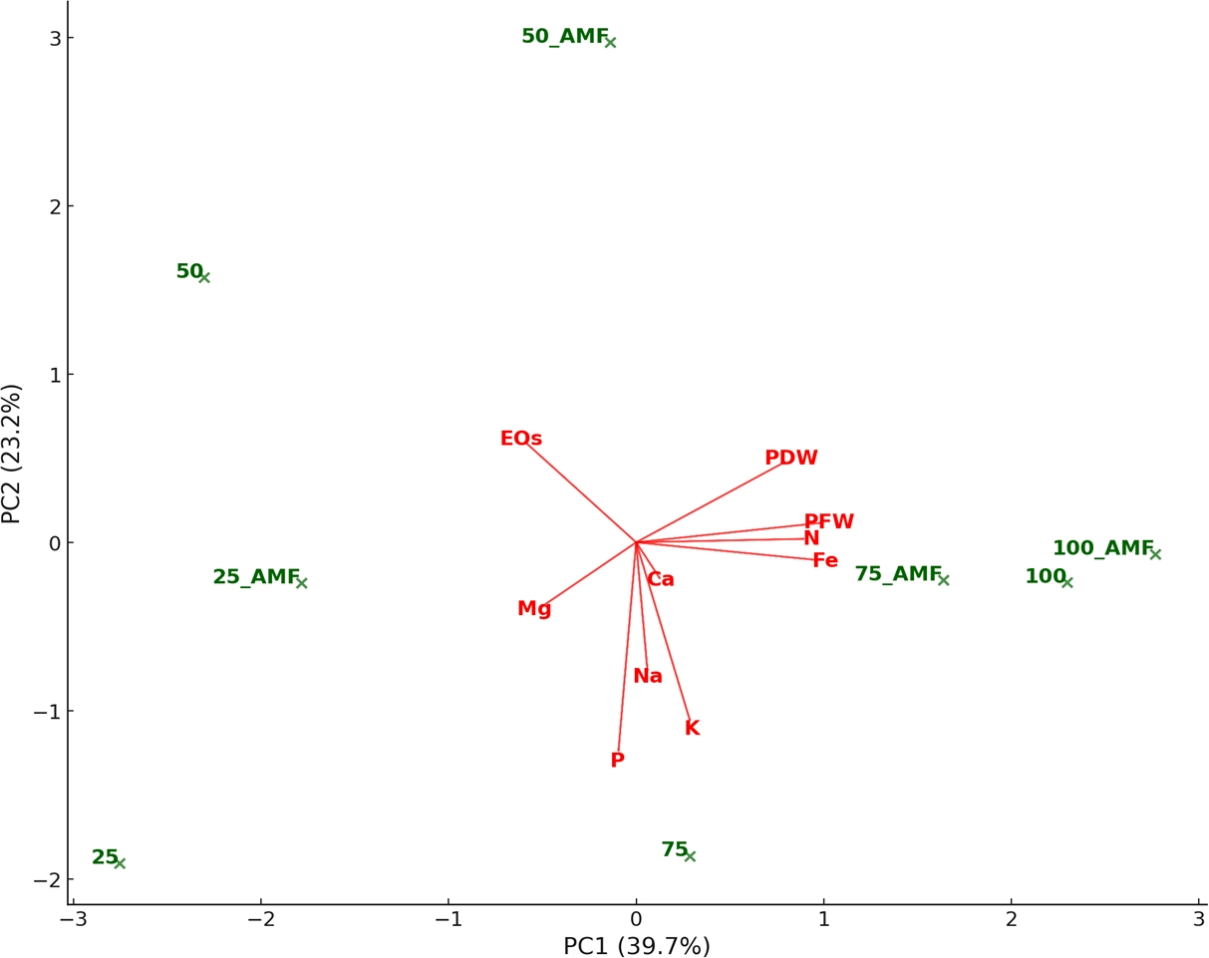
Principal component analysis biplot representing the relationships among treatments (IR25, IR50, IR75, IR100 with and without AMF application) and plant characteristics. Treatments (*i.e.,* observations) are plotted as green points. Variables are represented as red arrows. The length and direction of the arrows indicate the contribution of each variable to the first principal components (PC1 and PC2), which explain the primary patterns of variation in the dataset.

A principal component analysis (PCA) was performed to better visualize and interpret the relationships between variables and experimental conditions. Figure 10 represents the biplot obtained for PCA. The PC1 and PC2 were the principal axes of variation, effectively summarizing the dataset’s primary patterns. PC1 captured the largest share of variance, distinguishing treatments based on nutrient uptake and biomass traits, while PC2 accounted for additional variability linked to the interplay between water stress and AMF treatments. AMF treatments cluster near nutrient indicators such as N, P, and Mg, highlighting their role in improving nutrient absorption under diverse conditions. Higher 75% and 100% irrigation rates align closely with plant biomass metrics like plant fresh and dry weight, reflecting enhanced growth under optimal water availability. Conversely, 25% and 50% lower irrigation rates are positioned farther from biomass and nutrient variables, underlying the detrimental effects of water stress on growth and nutrient efficiency. Influenced by stress, essential EOs showed stronger associations with lower irrigation treatments.

## Discussion

The Mediterranean basin is one of the most drought-affected areas (Tramblay et al., 2020). Drought significantly limits plant growth, causing serious or fatal consequences, as water comprises 80–90% of plant biomass and plays a crucial role in growth and photosynthesis (Pradhan et al., 2020). MAPs are severely affected by drought, with substantial negative impacts on plant growth and productivity (Petropoulos et al., 2008). The present study investigated the effects of AMF application on drought mitigation in *O. onites* for the first time. Under drought stress, the fresh and dry weight evaluations showed that water limitation significantly reduced plant fresh and dry weights. In line with our findings, lower irrigation rates (*i.e.,* 88, 59, and 33%) decreased *O. onites* plant weights to 55 and 57%, respectively (Hancioglu et al., 2021). The same significant weight decreases were observed for two *O. vulgare* subspecies with 40 and 60% drought stress (Morshedloo et al., 2017). This behavior is typical in MAPs, which record reductions in vegetative growth under water limitation. For example, lavender (*Lavandula angustifolia*) is subjected to a water deficit for 30 days, which drastically decreases the plant’s fresh weight (Szekely-Varga et al., 2020). Similarly, sweet basil (*Ocimum basilicum*) cultivated for 43 days under three reduced levels of water supply (*i.e.,* 70, 50, and 30%) recorded fresh and dry weight losses of up to 56% (Mulugeta & Radácsi, 2022). Previous research on chamomile (*Matricaria chamomilla*) under varying irrigation regimes has reported that irrigation levels significantly affect plant dry weight and plant water-use efficiency (Baghalian et al., 2008; Pirzad et al., 2011; Pirzad et al., 2012; Salehi, Tasdighi & Gholamhoseini, 2016; Aleman & Marques, 2016). These observed growth constraints may be attributed to a reallocation of carbohydrates to favor root development or a reduction in photosynthetic efficiency under water imbalances (Hlahla et al., 2022). Water stress affects osmotic potential and stomatal aperture, reducing photosynthetic efficiency due to chlorophyll loss (Reza Yousefi et al., 2020). In prolonged drought, stomata close to conserve water, restricting CO_2_ entry, and leading to chlorophyll degradation. For medicinal and aromatic plants, water stress before flowering (vegetative stage) results in shorter plants with smaller leaf areas, as observed in genera such as *Mentha*, *Achillea*, *Calendula*, and *Melissa* (Gharibi et al., 2016; Ahmadi, Shabani & Sabzalian, 2019; Ghadyeh Zarrinabadi et al., 2019; Elansary et al., 2019). Reduced leaf area diminishes organic matter production due to lower photosynthetic efficiency (Tripathi et al., 2018).

Another aspect underlined by our results is the increase in the content of EOs in response to the decrease in irrigation level. Similar decreases were recorded for *O. onites* at medium and extreme water stresses (*i.e.,* 26–58%) and associated with aerial parts water loss (Hancioglu et al., 2021). Generally, drought stress induces the synthesis of secondary metabolites and EOs, which function as protective compounds against environmental challenges (Abdali et al., 2023). Recent studies have also highlighted the complex interplay between irrigation levels and the production of EOs in medicinal plants. A negative correlation between rainfall and *O. vulgare* EOs was observed, suggesting a role of these oils in plant adaptation to water stress (Napoli et al., 2020). Furthermore, adequate irrigation during seedling development and stem elongation, along with a reduction in water supply post-flowering, may enhance EOs content and improve the quality of oregano foliage (Azizi, Yan & Honermeier, 2009).

The irrigation level also significantly influenced plant nutrient contents. A literature survey showed that nutrient content changes under drought stress vary based on species. For example, a study carried out on six different MAPs (*i.e., Lavandula latifolia, Mentha piperita, Salvia sclarea, Salvia lavandulifolia, Thymus capitatus,* and *Thymus mastichina*) showed different nutrient changes. The leaf nitrogen concentration remained constant in *M. piperita*, *S. lavandulifolia*, *S. sclarea*, and *T. capitatus* during drought stress, but *L. latifolia* and *T. mastichina* exhibited a reduction in leaf nitrogen concentration under drought circumstances. Except for *S. sclarea,* P contents declined in all species. While no changes in leaf K concentrations (García-Caparrós et al., 2019). Significant reduction in leaf P concentrations was also reported in *Ocimum gratissimum* exposed to a weekly 78% water shortage (Osuagwu, Edeoga & Osuagwu, 2010). In all cases, nutrient decreases are linked to changes in root shape adaptation and uptake abilities in the presence of diverse water contents (Shoaib et al., 2022).

The application of AMF decreased the effects of water deficiency on growth (PFW and PDW). Similarly, AMF application has improved drought resistance in other MAPs, such as licorice (*Glycyrrhiza glabra*) (Fontana et al., 2009). The role of AMF in MAPs species drought stress mitigation is linked to the stimulation of plant responses at morphological, physiological, and molecular levels (Chandrasekaran, 2022; Israel et al., 2022). Key nutrient contents (K and N) were positively affected by AMF application, as also reported for lavender (Marulanda et al., 2007; Israel et al., 2022). The phylum Glomeromycota is known to establish symbiotic relationships with the roots of more than 80% of terrestrial plant species, including MAPs (Chen et al., 2018). Through this symbiosis, AMF enhances the uptake of water and essential nutrients such as phosphorus, nitrogen, and trace elements from the soil, while the fungi benefit from carbohydrates produced by the host plant (Harrier & Watson, 2003). This contrasts with P, Ca, Na, Mg, and Fe contents, for which an indirect involvement of AMF in the presence of water stress was observed (significance of AMF application factor only in the interaction with the irrigation level). This aspect should be further investigated. Nevertheless, it can be linked to the variability of water stress impact on nutrients, as discussed above, and the element differential solubility upon soil characteristics and root shape changes under drought stress (Zaman et al., 2024).

The AMF application factor was also significant for the accumulation of EOs only in the interaction with the irrigation level. The symbiosis established by AMF is recognized to be involved in alleviating the detrimental effects of drought conditions by several direct and indirect mechanisms (Israel et al., 2022). Based on the findings obtained in this study, the mechanisms involved seemed only indirect. Inoculation of *O. vulgare* with *Glomus viscosum*, for example, has been demonstrated to enhance the glandular density on the upper leaf epidermis, indirectly improving the content of EOs (Morone-Fortunato & Avato, 2008).

Correlation and PCA analyses underlined the interconnection among the investigated plant traits, nutrient dynamics, and AMF inoculation under drought stress. The positive correlation between plant fresh and dry weight with macronutrients such as N, P, and K confirms that improved nutrient uptake directly contributes to biomass accumulation (Chatzistathis et al., 2013). The AMF treatment correlation with key nutrient vectors (especially N and P) in the PCA biplot reinforces the role of *F. mosseae* in enhancing nutrient availability and assimilation (Bhupenchandra et al., 2024). Conversely, the inverse relationship between EOs content and biomass/nutrient uptake highlights a stress-induced secondary metabolite accumulation mechanism, commonly observed in MAPs under abiotic stress (Molinero-Ruiz & Chandrasekaran, 2022). The grouping of lower irrigation levels with EOs and sodium further reflects stress adaptation strategies, possibly *via* osmotic regulation or enhanced synthesis of protective compounds (Hancioglu et al., 2019).

By demonstrating the beneficial effects of *F. mosseae* inoculation in enhancing *O. onites* tolerance to drought stress, the study underscores the role of AMF as a sustainable practice in fostering resilience in MAPs against environmental stressors (Samantaray et al., 2024). Given the variability associated with AMF behavior in diverse pedological contexts (Delaeter et al., 2024). By adopting AMF-based cultivation practices, farmers can improve oregano resilience and profitability while reducing dependency on chemical inputs and irrigation resources. Therefore, our findings also add value to the role of microbial inoculants as effective mitigation strategies to alleviate the adverse effects of abiotic stresses in MAPs (Lenoir, Fontaine & Sahraoui, 2016). This sustainable approach is important given climate change’s increasing challenges, including extended drought and soil degradation events. Moreover, it is particularly relevant for Mediterranean regions, where oregano is extensively cultivated for commercial purposes (Reza Yousefi et al., 2020; Palmieri et al., 2021).

Recent studies have explored the viability of implementing AMF inoculation in field conditions, which presents key challenges (Berruti et al., 2016; Chen et al., 2024). AMF are obligate biotrophs, requiring a living host plant to survive and thrive (Gianinazzi et al., 2010). This presents a unique challenge in cultivating and maintaining AMF inocula at a large scale, as the production and application processes must be carefully managed to ensure the survival and proliferation of the fungi within the target crop’s root system (IJdo, Cranenbrouck & Declerck, 2011). While the long-term benefits of AMF in terms of improved nutrient acquisition, stress tolerance, and overall crop productivity are well-documented, the upfront costs associated with the procurement and application of AMF inocula may be a deterrent for some farmers, particularly those operating on tight budgets (Bitterlich et al., 2018). A multifaceted approach is required to address these challenges and unlock AMF’s full potential in field conditions. Ongoing research is focused on optimizing AMF production and formulation techniques and developing more efficient application methods that can be easily integrated into existing farming practices (Romero-Munar et al., 2023).

In this perspective, our results also contribute to adding knowledge to sustainable agriculture practices and commercial oregano production. The observed *F. mosseae* positive effects on *O. onites* tolerance to drought stress confirm the role of AMF as a sustainable practice in fostering resilience in MAPs against environmental stressors (Samantaray et al., 2024). Given the variability associated with AMF behavior in diverse pedological contexts (Delaeter et al., 2024), the findings add knowledge on the use of microbial inoculants as effective mitigation strategies to alleviate the adverse effects of abiotic stresses in MAPs (Lenoir, Fontaine & Sahraoui, 2016). This sustainable approach is important given climate change’s increasing challenges, including extended drought and soil degradation events. Moreover, it is particularly relevant for Mediterranean regions, where oregano is extensively cultivated for commercial purposes (Reza Yousefi et al., 2020; Palmieri et al., 2021).

## Conclusions

This study highlights the significant impact of irrigation levels and arbuscular mycorrhizal fungi (AMF) inoculation on an essential medicinal and aromatic plant’s growth, nutrient content, and stress resilience. Our findings suggest that the most effective and cost-efficient irrigation level for cultivating high-quality and productive *O. onites* with mycorrhizal inoculation is 75% irrigation. This level successfully maintained plant biomass and nutrient uptake comparable to full irrigation while enhancing essential oil yield due to moderate water stress conditions. Although our study did not investigate the complete physiological and molecular mechanisms of drought tolerance, the application of *F. mosseae* significantly improved plant growth. Our results demonstrate that while irrigation levels play a crucial role in determining plant fresh weight and nutrient content, AMF inoculation notably influences nutrient uptake, particularly for potassium (K), nitrogen (N), and iron (Fe). Despite variations in plant dry weight under different irrigation regimes, the incorporation of AMF significantly enhanced the growth parameters and nutrient assimilation of *O. onites*, underscoring its potential as a sustainable agricultural practice. The findings emphasize the importance of optimizing irrigation practices and leveraging AMF inoculation to mitigate the adverse effects of abiotic stresses on *O. onites*. Additional studies should be conducted to explore other AMF strains and their interactions with Turkish oregano under diverse environmental conditions. Further research is needed to elucidate the physiological and biochemical mechanisms underlying the improved drought tolerance in mycorrhizal plants. Moreover, the long-term effects of AMF inoculation on the sustainability of oregano production and the quality of EOs should be investigated further. Future research should focus on adjusting AMF application methods and exploring their interactions with different irrigation regimes to maximize their benefits in *O. onites* cultivation. Long-term field trials under diverse pedo-climatic contexts, exploration of other AMF strains, and the combination with other beneficial strains should also be further investigated. Nevertheless, the study is a valid scientific basis that confirms the potential of AMF inoculation as a suitable strategy to enhance the resilience of *O. onites* to drought stress. By improving nutrient uptake and growth performance, AMF inoculation enhances plant resilience and productivity under water scarcity. From the results of this initial study, new experiments can be developed on treating *O. onites* with AMF to improve tolerance to various environmental stresses. The AMF support sustainable agriculture and contribute to developing effective strategies for maintaining the productivity of MAPs amidst climate change and other environmental challenges.

##  Supplemental Information

10.7717/peerj.19499/supp-1 Supplemental Information 1 Raw data collection reporting irrigation rate, AMF treatment, plant fresh weight, plant dry weight, potassium, calcium phopshate, nitrogen magnesium, sodium, and iron content, essential oil content, fungal colonization and spore number

## Additional Information and Declarations

### Data Availability

The following information was supplied regarding data availability: The raw data are available in the Supplementary File.
